# Intensified post-stroke care improves long-term dysphagia recovery after acute ischemic stroke: Results from the STROKE CARD trial

**DOI:** 10.1177/23969873241284123

**Published:** 2024-10-10

**Authors:** Anel Karisik, Vincent Bader, Kurt Moelgg, Lucie Buergi, Benjamin Dejakum, Silvia Komarek, Christian Boehme, Thomas Toell, Lukas Mayer-Suess, Simon Sollereder, Sonja Rossi, Patricia Meier, Gudrun Schoenherr, Johann Willeit, Peter Willeit, Wilfried Lang, Stefan Kiechl, Michael Knoflach, Raimund Pechlaner

**Affiliations:** 1 Department of Neurology, Medical University of Innsbruck, Innsbruck, Austria; 2 VASCage – Centre on Clinical Stroke Research, Innsbruck, Austria; 3 ICONE – Innsbruck Cognitive Neuroscience, Department for Hearing, Speech and Voice Disorders, Medical University of Innsbruck, Innsbruck, Austria; 4 Institute of Clinical Epidemiology, Public Health, Health Economics, Medical Statistics and Informatics, Medical University of Innsbruck, Innsbruck, Austria; 5 Department of Public Health and Primary Care, University of Cambridge, Cambridge, UK; 6 Medical Faculty, Sigmund Freud Private University, Vienna, Austria

**Keywords:** Dysphagia, ischemic stroke, recovery, intensified care

## Abstract

**Introduction:**

Dysphagia is common after acute ischemic stroke and entails considerable morbidity and mortality. Here, we investigated the impact of intensified care on swallowing recovery after stroke.

**Patients and methods:**

In this secondary analysis of STROKE-CARD, a randomized intervention trial of intensified post-stroke care, dysphagia was assessed by speech therapists at admission for acute ischemic stroke, at hospital discharge, and after 12-months. Patients randomized to STROKE-CARD care additionally received a detailed dysphagia follow-up at 3-months, including a standardized dysphagia examination, instructions on further exercises and compensation mechanisms and, if necessary, referral for further speech therapy.

**Results:**

Dysphagia was present initially after stroke in 236 (16.6%; median age 82 (73–88), 44.1% female) of 1419 patients, with similar prevalence in both study groups at hospital admission (*p* = 0.239) and discharge (*p* = 0.870). At follow up, 14 (9.5%) of 147 in the intervention group and 18 (20.2%) of 89 in the control group suffered from persistent dysphagia (*p* = 0.020). There was better dysphagia recovery in the intervention group also under multivariable adjustment for age, sex, functional disability at 12-months, severe dysphagia at hospitalization, mode of feeding, cognitive impairment, thrombolysis, and stroke localization (odds ratio, 0.41, 95% confidence interval: 0.17 to 0.96).

**Discussion and conclusion:**

Intensified post-stroke care improved dysphagia recovery within 1 year after acute ischemic stroke, highlighting the potential of targeted interventions for enhancing stroke outcomes.

## Graphical abstract

**Graphical abstract:**
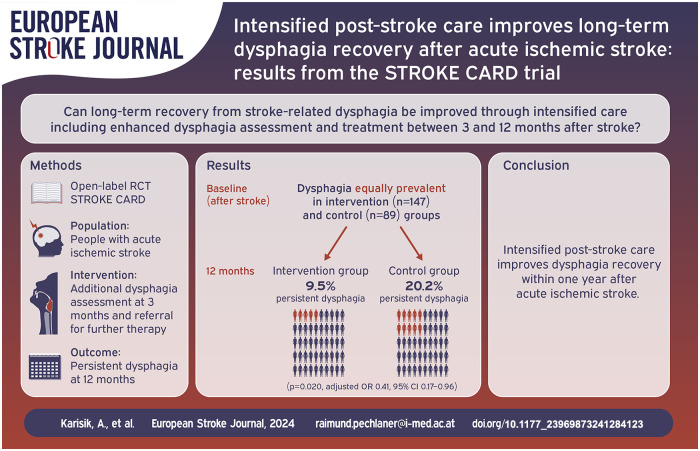

## Introduction

Dysphagia ranks among the most common complications after acute ischemic stroke and impacts considerably on functional outcome and quality of life.^1^ Although swallowing function often recovers within weeks or months, some patients suffer from persistent dysphagia. Prevalence of dysphagia decreases from 51 to 86% 1 week after stroke to 29 to 40% in the first to third month, and in 8 to 13% dysphagia persists after 6 months.^2–6^ Dysphagia affects functional outcome and quality of life also by predisposing to anxiety and depression, particularly when it is persistent.^7^

Although research on therapies for post-stroke dysphagia is advancing rapidly,^1,8,9^ most approaches focus on the acute and subacute phase after stroke. Here we investigated how dysphagia recovery may be aided on a long run through an intensified post-stroke care program, STROKE-CARD.

## Methods

STROKE-CARD is a randomized clinical trial (Clinical Trials.gov registration number: NCT02156778) that was conducted between 2014 and 2017 at Innsbruck University Hospital, Innsbruck, Austria, and St. John of God Hospital, Vienna, Austria, that showed the efficacy of intensified post-stroke care for reducing cardiovascular events and improving health-related quality of life after acute ischemic stroke or high-risk transient ischemic attack (ABCD_2_ score ⩾ 3).^10^ Patients aged at least 18 years were enrolled and randomly allocated to either STROKE-CARD care or standard care. Exclusion criteria included malignancy, a life expectancy of less than 1 year, drug addiction, alcohol abuse, residence outside the hospital’s catchment area, or severe disability as indicated by a modified Rankin Scale score of 5 at hospital discharge.^10,11^ Regardless of having dysphagia, patients were randomized in blocks of alternating 8- and 4-week durations in a 2:1 ratio to either STROKE-CARD or standard care at hospital discharge (median hospital stay was 9 days, IQR 6–13).^10,11^

The STROKE-CARD intervention included a 3-month outpatient visit performed by a multidisciplinary team including physicians, nurses, physiotherapists, and occupational and speech/language therapists. In addition to structured stroke aftercare including risk adjustment and drug therapy optimization, a thorough screening for post-stroke complications and the identification of persistent stroke deficits and further therapy requirements was carried out.^10,11^

### Dysphagia-specific intervention

A clinical swallowing examination was performed at 3 months after stroke in all patients that experienced dysphagia during hospital stay and survived until 3 months. The standardized examination was carried out by an interdisciplinary team of speech therapists and neurologists. Patients that experienced persistent dysphagia at 3 months were trained in further swallowing exercises, to be performed in the outpatient setting, and were trained in compensation mechanisms to aid effective swallowing. In addition, therapy status and need for further speech and swallowing therapy were assessed and further outpatient or inpatient therapy was initiated where necessary.

### Study population

**Figure 1.**
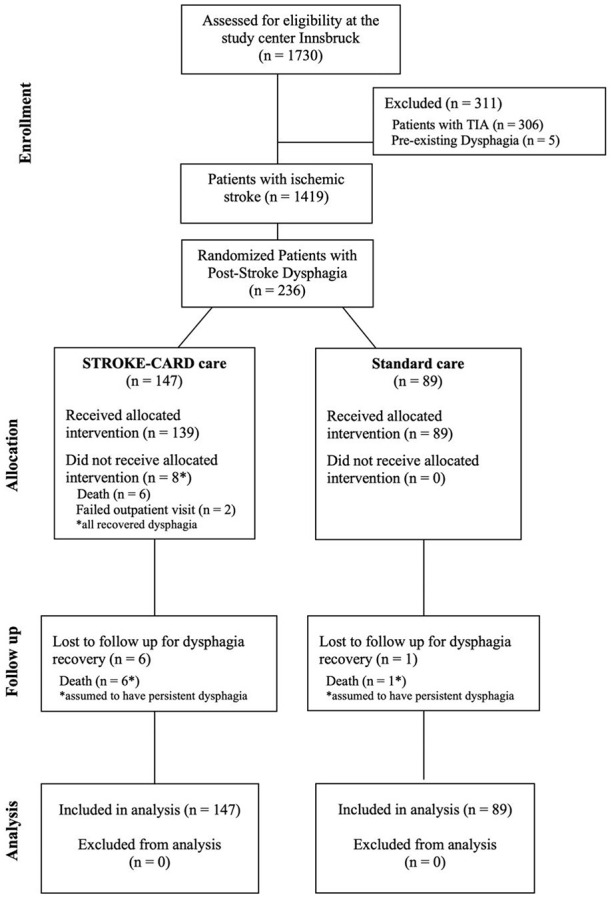
Study flow chart.

Data on dysphagia were reported solely from the study center Innsbruck (Austria). Of 2149 patients enrolled at both study centers, 1730 patients were recruited in Innsbruck. After excluding 306 patients presenting with TIA (tissue-based definition) and 5 patients with pre-existing dysphagia, a total of 1419 patients with ischemic stroke remained for further assessment. Dysphagia was present in 236 patients at hospitalization, of which 147 were allocated to STROKE-CARD care and 89 to standard care (Figure 1).

The Stroke-Card Study received approval from the Medical University of Innsbruck’s ethics committee. All necessary consent from patients was obtained and required institutional documents were properly archived.

### Swallowing assessment and Outcome measures

As part of routine clinical care all patients were screened for swallowing difficulties by an experienced team including nurses, speech and language therapists, and physicians at hospitalization. Dysphagia was diagnosed via clinical examination of swallowing function by speech and language therapists supplemented by further diagnostic approaches such as fiberoptic endoscopic evaluation of swallowing (FEES) where indicated. Therapists did not have access to medical documentation informing on study group allocation of patients, but no formal blinding procedure was implemented for swallowing assessment and only the intervention group received a 3-month follow-up visit.

The need for alternative feeding by nasogastric tube (NGT) or percutaneous endoscopic gastrostomy (PEG) during hospitalization was recorded. The severity of swallowing dysfunction was graded according to nutritional recommendations based on the recorded swallowing examinations into mild (prohibition of mixed consistencies), moderate (prohibition of liquids without thickening), and severe (no oral nutrition).

In patients with initial dysphagia, the first assessment was used as baseline assessment. Swallowing function was reassessed during hospitalization and at hospital discharge, and in case of allocation to STROKE-CARD care, at the 3-month intervention. All patients received a follow-up clinical swallowing examination at 12-month follow-up. The main outcome at 12-months was defined as return to full oral diet and no need for further dysphagia therapy. Loss to follow-up before 12-months after stroke was mainly due to death (Figure 1). Potential bias arising from loss to follow-up was mitigated by reviewing detailed speech therapy reports created during inpatient and outpatient rehabilitation for dysphagia recovery, as indicated by resumption of unrestricted oral food intake and discontinuation of speech therapy. A last observation carried forward approach was used as main analysis, while sensitivity analyses examined results when including only patients that appeared at the 12-month follow-up.

Stroke was categorized as anterior circulation or posterior circulation stroke, with severity of the neurological deficit graded by National Institute of Health Stroke Severity Scale NIHSS^12^ and functional disability by modified Rankin Scale mRS^13^ at admission, discharge and 3- and 12-month follow-ups, dysarthria at admission, cognitive impairment (documented in the digital health record or diagnosed using the Mini-Mental State Exam with a score < 25 at baseline) and pneumonia diagnosis during the first year (as documented in the digital health record).

### Statistical analysis

Participant characteristics are shown as median (interquartile range) or count (percentage). Group comparisons of STROKE-CARD versus standard care were performed using Mann-Whitney *U* tests for continuous variables and Pearson’s chi-squared test for categorical variables. None of the continues variables was normally distributed according to Shapiro-Wilk tests. Bonferroni adjustment addressed multiple testing in univariate comparisons. Missing data were managed by complete case analysis und numbers of missing values are shown in tables. Additionally, the impact of STROKE-CARD care on swallowing recovery was assessed using multivariable logistic regression including adjustment for age, sex, and severe dysphagia at hospital admission (model 1), for these variables in addition to cognitive impairment, alternative feeding, functional disability (mRS) at 12-months, thrombolysis, posterior circulation and bilateral stroke (model 2), or for the variables included in model 1 in addition to dysarthria at admission, cognitive impairment, alternative feeding, stroke severity (NIHSS) at baseline, thrombolysis, posterior circulation, and bilateral stroke (model 3). Sensitivity analyses assessed the impact of loss to follow-up by considering two extreme scenarios (assuming that among patients lost to follow-up, none belonging to the intervention group but all belonging to the control group had suffered from persistent dysphagia – Extremum 1, or vice versa – Extremum 2). Additional analyses limiting the cohort to those with persistent dysphagia at hospital discharge is provided in the supplementary material (Table 1). An alpha level of 0.05 is used. Analyses were performed using SPSS Version 27.0.1.0 (IBM Corporation, Armonk, New York).

**Table 1.** Characteristics of the study population.

Characteristic	All patients (*n* = 236)	STROKE-CARD care (*n* = 147)	Standard care (*n* = 89)	*p* Value*
	*n* (%) or median (IQR)			
Baseline characteristics				
Female sex	104 (44.1%)	67 (45.6%)	37 (41.6%)	0.548
Age	82 (73–88)	82 (73–88)	82 (73–88)	0.683
Body mass index	26 (24–29)	26 (23–29)	26 (24–29)	0.334
Arterial hypertension	201 (85.2%)	123 (83.7%)	78 (87.6%)	0.406
Dyslipidemia	195 (82.6%)	120 (81.6%)	75 (84.3%)	0.604
Diabetes	54 (22.9%)	31 (21.1%)	23 (25.8%)	0.399
Atrial fibrillation	85 (36.0%)	51 (34.7%)	34 (38.2%)	0.586
Cognitive impairment	18 (7.6%)	10 (6.8%)	8 (9.0%)	0.540
Stroke territory				
Anterior circulation	157 (66.5%)	106 (72.1%)	51 (57.3%)	0.019
Posterior circulation	68 (28.8%)	34 (23.1%)	34 (38.2%)	0.013
Both	11 (4.7%)	7 (4.8%)	4 (4.5%)	0.925
Lesion localization				
Unilateral left	119 (50.4%)	69 (46.9%)	50 (56.2%)	0.169
Unilateral right	94 (39.8%)	64 (43.5%)	30 (33.7%)	0.135
Bilateral lesion	23 (9.7%)	14 (9.5%)	9 (10.1%)	0.883
Outcome measures at baseline				
NIHSS, admission	7 (4–12)	6 (4–12)	7 (4–13)	0.962
mRS, admission	4 (3–4)	4 (3–5)	4 (3–4)	0.713
Thrombolysis	79 (33.5%)	55 (37.4%)	24 (27.0%)	0.099
Thrombectomy	25 (10.6%)	17 (11.6%)	8 (9.0%)	0.533
Dysarthria, admission	120 (50.8%)	76 (51.7%)	44 (49.4%)	0.736
NIHSS, discharge	3 (1–6)	3 (1–6)	3 (1–5)	0.071
mRS, discharge	3 (2–4)	3 (2–4)	3 (2–3)	0.284
Discharged to nursing home	8 (3.4%)	4 (2.7%)	4 (4.5%)	0.466
Discharged to rehabilitation	135 (57.2%)	88 (59.9%)	47 (52.8%)	0.288

IQR: interquartile range; mRS: modified Rankin Scale; NIHSS: National Institutes of Health Stroke Scale.

* *p* Values for differences between STROKE-CARD care and standard care. The significance threshold adjusted for multiple testing using the Bonferroni method is *p* ⩽ 0.002.

## Results

Of 1419 patients with ischemic stroke, 236 (16.6%) were diagnosed with dysphagia at hospitalization and either allocated to STROKE-CARD care (*n* = 147) or standard care (*n* = 89) (Figure 1).

Median age at baseline was 82 (interquartile range: 73–88) years and 44.1% of study participants were female. There was a preponderance of anterior circulation (66.5%) and left-sided stroke (50.4%), and 33.5% of patients received thrombolysis (Table 1).

Study groups were similar at baseline except for a relative preponderance of posterior circulation stroke in the control group (Table 1). Importantly, there were no significant differences in dysphagia prevalence or severity between study groups (Table 2), and no significant differences in initial stroke severity according to NIHSS and mRS scores at admission and discharge (Table 1).

**Table 2.** Dysphagia characteristics at baseline.

Characteristic	All patients	STROKE-CARD care	Standard care	*p* Value*
	% (*n*) or median (IQR)			
Dysphagia characteristics				
Hospitalization				
Dysphagia	16.6% (236/1419)	15.8% (147/929)	18.2% (89/490)	0.239
Mild	52.1% (123/236)	55.8% (82/147)	46.1% (41/89)	
Moderate	38.1% (90/236)	37.4% (55/147)	39.3% (35/89)	0.104
Severe	9.7% (23/236)	6.8% (10/147)	14.6% (13/89)	
FEES	25.0% (59/236)	24.5% (36/147)	25.8% (23/89)	0.816
NGT/PEG	7.6% (18/236)	6.1% (9/147)	10.1% (9/89)	0.263
Intubation	4.7% (11/236)	5.4% (8/147)	3.4% (3/89)	0.464
Hospital discharge				
Dysphagia	13.3% (189/1419)	13.4% (125/929)	13.1% (64/490)	0.870

FEES: fiberoptic endoscopic evaluation of swallowing; NGT: nasogastric tube; PEG: percutaneous endoscopic gastrostomy.

* *p* Values for the differences between STROKE-CARD care and standard care.

Of 236 patients with dysphagia at hospitalization, 52.1% showed mild, 38.1% moderate, and 9.7% severe swallowing impairment (Table 2). 25.0% of patients received a FEES in addition to clinical swallowing examination. Alternative feeding during hospitalization via NGT or PEG was performed in 7.6%, and intubation and mechanical ventilation in 4.7% (Table 2). At hospital discharge, dysphagia persisted in 13.3% (189 out of 1419) of patients overall, and in 13.4% and 13.1% of patients allocated to STROKE-CARD and standard care (*p* = 0.870).

All patients with persistent dysphagia at discharge either received the allocated intervention (*n* = 139) or were lost to follow-up (8 patients missed the 3-month visit, 6 of which because they had died) after complete recovery of swallowing function had been documented. At the 3-month visit, dysphagia was present in 23% (32/139) of patients with initial dysphagia, which resulted in 10 referrals to additional outpatient speech and language therapy and 8 referrals to inpatient rehabilitation including speech and language therapy.

Follow-up at 12-months was 97% complete, with 229 of 236 included patients attending. Of seven patients that died with persistent dysphagia before 12-months, 6 had received STROKE-CARD care and 1 standard care, and main analyses include these seven in the persistent dysphagia at 12-months group using a last observation carried forward approach.

Dysphagia was present in 13.6% of patients (32 out of 236) at 12-months. Patients receiving STROKE-CARD care were significantly less likely to have persistent dysphagia than those receiving standard care (9.5% vs 20.2%, *p* = 0.020). Overall functional outcome as assessed by mRS at 12-months did not significantly differ between study groups (*p* = 0.446). Pneumonia incidence after discharge was nominally lower in the intervention group (4.8% vs 9.0%, *p* = 0.197; Table 3).

**Table 3.** Impact of STROKE-CARD care on dysphagia recovery.

Characteristic	Standard care (*n* = 89)	STROKE-CARD care (*n* = 147)	*p* Value
Primary outcome – Univariable	Prevalence, %		
Persistent dysphagia at follow-up	18 (20.2%)	14 (9.5%)	0.020
Primary outcome – Multivariable	Odds ratio (95% CI)		
Model 1	1.00 (ref.)	0.43 [0.20, 0.92]	0.029
Model 2	1.00 (ref.)	0.41 [0.17, 0.96]	0.039
Model 3	1.00 (ref.)	0.43 [0.18, 0.99]	0.049
Sensitivity analyses	Odds ratio (95% CI)		
Model 1 – Extremum 1	1.00 (ref.)	0.23 [0.10, 0.57]	0.001
Model 1 – Extremum 2	1.00 (ref.)	0.46 [0.21, 0.99]	0.049
Secondary outcomes – Univariable	Prevalence, % or median (IQR)		
Pneumonia after discharge	8 (9.0%)	7 (4.8%)	0.197
mRS, 12-months	3 (1,4)	3 (2,4)	0.446

OR: odds ratio; CI: confidence interval; IQR: interquartile range; mRS: modified Rankin Scale; NIHSS: National Institutes of Health Stroke Scale.

All patients had dysphagia at baseline. Odds ratios are for the association of study group with dysphagia at 12 months follow-up. Before the 12-month follow-up, 6 patients in the intervention group and 1 patient in the control group died. The main analysis carried the last observation forward for these patients. Sensitivity analyses addressed follow-up loss by assuming either none in the intervention and all in the control group had persistent dysphagia (Extremum 1) or the opposite (Extremum 2). Model 1: Adjustment for age, sex, and severe dysphagia at hospital admission. Model 2: As model 1, with additional adjustment for cognitive impairment, alternative feeding, functional disability (mRS) at 12-months, thrombolysis, posterior circulation and bilateral stroke. Model 3: As model 1, with additional adjustment for dysarthria at admission, cognitive impairment, alternative feeding, stroke severity (NIHSS) at baseline, thrombolysis, posterior circulation and bilateral stroke.

After multivariable adjustment for either age, sex, severe dysphagia at hospital admission (model 1) and for age, sex, severe dysphagia at hospital admission, cognitive impairment, posterior circulation stroke, bilateral lesion, thrombolysis, initial need for alternative feeding and functional disability at 12-months (model 2), STROKE-CARD care remained independently associated with a lower prevalence of persistent dysphagia at 12-months (Model 1: OR 0.43 95% CI [0.20, 0.91], *p* = 0.029; Model 2: OR 0.41 95% CI [0.17, 0.96], *p* = 0.039). Substituting functional disability at 12-months with initial stroke severity (NIHSS at admission) and adding dysarthria to the model did not appreciably change results (model 3). In addition, sensitivity analyses assessed the impact of loss to follow-up by considering two extreme scenarios (Table 3) and limiting the cohort to only those with persistent dysphagia at hospital discharge (Table S1) yielded comparable results. Lastly, including thrombectomy in the models did not change the outcome (data not shown).

## Discussion

In this analysis of the STROKE-CARD trial, which evaluated the benefits of intensified post-stroke care, we investigated the effect of targeted interventions on swallowing recovery in patients with dysphagia 1 year after acute ischemic stroke. Patients who received the STROKE-CARD intervention were significantly more likely to recover swallowing function compared to standard care.

The STROKE-CARD intervention included a structured, multidisciplinary follow-up swallowing examination at 3-months after stroke providing further instructions for swallowing exercises and compensation mechanisms and, in addition, referral to further in- or outpatient speech and language rehabilitation if needed. To the best of our knowledge, this is the first report of improved swallowing recovery within 1 year after stroke through intensified, targeted post-stroke care.

The association of intensified care with swallowing recovery was robust to adjustment for important covariates that have previously been identified as predictors for persistent dysphagia, including stroke-related characteristics such as severity (NIHSS) or functional disability (mRS), stroke localization (anterior vs posterior circulation and specific brain regions involved), right hemisphere and bilateral brain lesions.^3,6,14–19^ Furthermore, factors related to swallowing difficulties, such as dysphagia severity, aspiration risk, prolonged oral transit time, and intubation, beside others like older age, lower body mass index, larger white matter hyperintensity volume, and cognitive decline, were also found to be associated with persistent dysphagia.^3,4,6,15–18,20,21^ In contrast, younger age and left-hemisphere stroke were associated with better recovery in those receiving PEG.^22^

These results suggest that the STROKE-CARD intervention aids swallowing recovery independently of known predictors of persistent dysphagia after stroke and, most importantly, independent of general functional recovery as indicated by the modified Rankin Scale.

In this large cohort of patients with ischemic stroke, dysphagia was diagnosed in 16.6% of patients. Reported prevalences of dysphagia after stroke are highly variable because of differences in dysphagia assessment: 13–49% in studies that used screening tests or clinical swallowing examinations versus 64–78% in studies that used instrumental approaches like fiberoptic endoscopy, and study populations, which partly also included hemorrhagic strokes.^23,24^ Another potential reason for the low prevalence may be attributable to excluding patients with severe (mRS ⩾ 5) stroke outcome at hospital discharge.

Among those initially diagnosed with dysphagia, it persisted until hospital discharge in 80% of cases, with 13% still experiencing dysphagia at 12-months, which closely aligns with previous reports of 51–86% 1 week after stroke and 8–13% 6 months after.^2–6^

Strengths of this study include that it reflects a broad spectrum of patients with acute ischemic stroke, such that findings likely apply to unselected stroke patients. Dysphagia was diagnosed throughout all study timepoints via clinical swallowing examination by experienced speech and language therapists. All patients with persistent dysphagia at hospital discharge and allocated to STROKE-CARD care received the 3-month intervention if alive at 3-months. Furthermore, loss to follow-up was low with 97% of included patients completing the 12-month follow-up. The few patients who died before 12-months with persistent dysphagia, analyzed as if dysphagia had persisted until the 12-month assessment. As such patients were more numerous in the intervention group, results of our main analysis are expected to be conservative. Excluding those who died from our analysis further strengthened the association between the intervention and dysphagia recovery.

One limitation is that patients with severe disability, as indicated by a modified Rankin Scale score of 5 at hospital discharge, were not included in STROKE CARD, although they may be predisposed to dysphagia. Low overall counts of subjects with persistent dysphagia at 12-months precluded subgroup analysis. Moreover, no validated scores were available to grade dysphagia severity. Finally, the rate of mechanical thrombectomy may have shifted our cohort toward relatively milder outcomes of dysphagia.

The current study, which primarily evaluated the effect of a comprehensive disease management program on a blinded cardiovascular outcome, involved intensified dysphagia screening and treatment with an unblinded dysphagia outcome. A more focused intervention study dedicated wholly to dysphagia recovery might indicate even greater benefits, which remains for future studies to investigate.

## Conclusion

A targeted post-stroke intervention improved swallowing recovery at 1 year after acute ischemic stroke, highlighting the potential of intensified post-stroke care for dysphagia recovery on a long run.

## Supplemental Material

sj-docx-1-eso-10.1177_23969873241284123 – Supplemental material for Intensified post-stroke care improves long-term dysphagia recovery after acute ischemic stroke: Results from the STROKE CARD trial Supplemental material, sj-docx-1-eso-10.1177_23969873241284123 for Intensified post-stroke care improves long-term dysphagia recovery after acute ischemic stroke: Results from the STROKE CARD trial by Anel Karisik, Vincent Bader, Kurt Moelgg, Lucie Buergi, Benjamin Dejakum, Silvia Komarek, Christian Boehme, Thomas Toell, Lukas Mayer-Suess, Simon Sollereder, Sonja Rossi, Patricia Meier, Gudrun Schoenherr, Johann Willeit, Peter Willeit, Wilfried Lang, Stefan Kiechl, Michael Knoflach and Raimund Pechlaner in European Stroke Journal
