# Supplementary material for: Intensified post-stroke care improves long-term dysphagia recovery after acute ischemic stroke: Results from the STROKE CARD trial
Source: Eur Stroke J. 2024 Oct 10;10(2):568–75. doi: 10.1177/23969873241284123 (PMC11556674; doi:10.1177/23969873241284123)
Supplement: sj-docx-1-eso-10.1177_23969873241284123 – Supplemental material for Intensified post-stroke care improves long-term dysphagia recovery after acute ischemic stroke: Results from the STROKE CARD trial [file sj-docx-1-eso-10.1177_23969873241284123.docx]

**SUPPLEMENTARY MATERIAL**

**Intensified post-stroke care improves long-term dysphagia recovery after acute ischemic stroke: results from the STROKE CARD trial**

Anel Karisik, MD^1,2^, Vincent Bader^2^, Kurt Moelgg, MD^1,2^, Lucie Buergi, MD^1,2^, Benjamin Dejakum, MD^2^, Silvia Komarek, MD^2^, Christian Boehme, MD, PhD^2^, Thomas Toell, MD, PhD^2^, Lukas Mayer-Suess, MD, PhD^2^, Simon Sollereder, MSc^1^, Sonja Rossi, PhD^3^, Patricia Meier, MSc^1^, Gudrun Schoenherr, MSc^2^, Johann Willeit, MD^2^, Peter Willeit, MD, PhD^4,5^, Wilfried Lang, MD^1,6^, Stefan Kiechl, MD^1,2^, Michael Knoflach, MD^1,2^, Raimund Pechlaner, MD, PhD^2^ for the STROKE-CARD study group.

^1^VASCage – Centre on Clinical Stroke Research, Innsbruck, Austria
^2^Department of Neurology, Medical University of Innsbruck, Innsbruck, Austria
^3^ICONE - Innsbruck Cognitive Neuroscience, Department for Hearing, Speech and Voice Disorders, Medical University of Innsbruck, Innsbruck, Austria
^4^Institute of Health Economics, Medical University of Innsbruck, Innsbruck, Austria
^5^Department of Public Health and Primary Care, University of Cambridge, Cambridge, UK
^6^Medical Faculty, Sigmund Freud Private University, Vienna, Austria

Corresponding Author: Priv.-Doz. Dr. Raimund Pechlaner, PhD, Department of Neurology, Medical University of Innsbruck, Anichstr. 35, 6020 Innsbruck, Austria, Phone: +43-512-504-82914, Email: raimund.pechlaner@i-med.ac.at

**SUPPLEMENTAL MATERIAL:**

Table 1. Impact of STROKE-CARD care on dysphagia recovery (only patients with persistent dysphagia at hospital discharge).

|  |  | **Standard care  (n=64)** | **STROKE-CARD care**  **(n=125)** |  |
| --- | --- | --- | --- | --- |
| **Univariable** |  | **Prevalence, %** | | **p value** |
| **Persistent dysphagia at follow-up** |  | 18 (28.1%) | 14 (11.2%) | 0.003 |
| **Multivariable** |  | **Odds ratio (95% CI)** | | **p value** |
| Model 1 |  | 1.00 (ref.) | 0.30 [0.13, 0.67] | 0.003 |
| Model 2 |  | 1.00 (ref.) | 0.23 [0.09, 0.60] | 0.003 |
| Model 3 |  | 1.00 (ref.) | 0.32 [0.13, 0.78] | 0.012 |
| **Sensitivity Analyses** |  | **Odds ratio (95% CI)** | | **p value** |
| Model 1 – Extremum 1 |  | 1.00 (ref.) | 0.16 [0.06, 0.41] | <0.001 |
| Model 1 – Extremum 2 |  | 1.00 (ref.) | 0.32 [0.14, 0.73] | 0.006 |
| All patients had dysphagia at baseline. Odds ratios are for the association of study group with dysphagia at 12 months follow-up.  Before the 12 month follow-up, 6 patients in the intervention group and 1 patient in the control group died. The main analysis carried the last observation forward for these patients. Sensitivity analyses give bounds on the uncertainty due to loss to follow-up by providing effects when assuming that among patients lost to follow-up, none belonging to the intervention group but all belonging to the control group had suffered from persistent dysphagia (Extremum 1), or vice versa (Extremum 2).  OR - Odds Ratio, CI - Confidence interval, mRS - modified Rankin Scale, NIHSS - National Institutes of Health Stroke Scale  Model 1: Adjustment for age, sex, and severe dysphagia at hospital admission.  Model 2: As model 1, with additional adjustment for cognitive impairment, alternative feeding, functional disability (mRS) at 12 months, thrombolysis, posterior circulation and bilateral stroke.  Model 3: As model 1, with additional adjustment for dysarthria at admission, cognitive impairment, alternative feeding, stroke severity (NIHSS) at baseline, thrombolysis, posterior circulation and bilateral stroke. | | | | |
